# Norm Emergence through Conflict-Blocking Interactions in Industrial Internet of Things Environments

**DOI:** 10.3390/s24186047

**Published:** 2024-09-19

**Authors:** Yuchen Wang, Yanqin Miao, Gang Fu, Peng Lu, Yikun Yang, Wen Gu, Zijie Fang, Lei Niu

**Affiliations:** 1 School of Civil Engineering, Guangzhou University, Guangzhou 510006, China; 2 Shaoxing Electric Power Equipment Co., Ltd., Shaoxing 312025, China; 3 School of Information and Electronic Engineering, Zhejiang University of Science and Technology, Hangzhou 310023, China; 4 Institute of Computing Innovation, Zhejiang University, Hangzhou 311200, China; 5 School of Computing and Information Technology, University of Wollongong, Wollongong, NSW 2522, Australia; 6 Japan Advanced Institute of Science and Technology, Nomi 923-1292, Japan; 7 School of Computer Science, Faculty of Engineering and Information Technology, University of Technology Sydney, Sydney, NSW 2007, Australia; 8 Faculty of Artificial Intelligence in Education, Central China Normal University, Wuhan 430079, China

**Keywords:** norms, decentralized norm emergence, coordination, industrial internet of things

## Abstract

Norms have been effectively utilized to facilitate smooth interactions among agents. Norms are usually the global data that agents cannot directly access in complex environments; instead, norms can only be indirectly accessed by agents via maintaining their own beliefs about norms. Establishing norms using decentralized interaction-based methods has attracted much attention. However, the current methods overlook Industrial Internet of Things (IIoT) environments. In IIoT, there is a prevalent feature called “conflict-blocking”, where agents’ conflicting action strategies can block an interaction from being completed or even cause danger. To facilitate norm emergence in IIoT, we propose a framework to support agent decisions in conflict-blocking interactions. The framework aids in achieving system scalability by integrating the fusion of agent beliefs about norms. We prove that the proposed framework guarantees norm emergence. We also theoretically and experimentally analyze the time required for norm emergence under the influence of various factors, such as the number of agents. A vehicle movement simulator is also developed to vividly illustrate the process of norm emergence.

## 1. Introduction

Norms are essential for guiding human behavior and ensuring societal stability. When individuals adhere to norms, they engage in predictable behavior strategies during social interactions, expecting reciprocal adherence from others [1]. When everyone conforms to a norm, coordinated strategies ensure smooth interactions.

Norms can be established through either centralized or decentralized methods [2]. Centralized methods involve a central controller informing each agent of the strategy to adopt, which is often impractical due to high costs or infeasibility [3]. Consequently, decentralized methods, often referred to as norm emergence, are more extensively studied. Norm emergence refers to the process by which agents gradually adopt consistent strategies in interactions and relies on decentralized agent interactions occurring without central control. During these interactions, agents may adjust strategies when encountering uncoordinated strategies. Through repeated interactions and strategy adjustments, a norm might emerge. For simplicity, we use “conflict” to indicate uncoordinated strategies.

The current methods for norm emergence overlook Industrial Internet of Things (IIoT) environments. In IIoT, there is a prevalent feature called “conflict-blocking”, where agents’ conflicting strategies can block an interaction from being completed or even cause danger [4]. For instance, when two vehicles approach each other, the vehicles need to pass each other, i.e., to finish a passing interaction. The interaction is considered “blocked” if the vehicles’ paths overlap (conflict), leading to a potential collision. A blocked interaction disrupts the normal function of the vehicles, which is undesirable. If a norm emerges, such as all vehicles moving on the right side or left side of the road, efficient passing without disruption can be achieved.

However, enabling norm emergence in IIoT environments faces significant challenges. First, in IIoT, agents usually do not have direct access to global data relating to norms such as which strategy is used by the majority. The lack of global visibility causes difficulty for agents in making informed decisions in terms of establishing norms, leading to inconsistent and potentially conflicting strategies. Second, achieving system scalability while ensuring that norms can emerge efficiently is challenging. This is due to the dynamic nature of the IIoT where agents may continuously join and leave a system. Newly joined agents may not conform to existing norms. The adjustments of strategies to maintain smooth interactions disrupts established norms, reducing system efficiency. Third, the heterogeneous nature of IIoT agents means that agents may have different objectives and costs associated with strategy adjustments. Such heterogeneity could lead to situations where, e.g., some agents act in self-interest, disregarding the collective need for norm emergence.

To address these challenges, we propose a framework to support agent decisions in conflict-blocking interactions. The framework integrates the fusion of agent beliefs about norms with local data. The framework focuses on three agent desires to deal with agent heterogeneity: resolving conflicts to maintain normal function, enabling norm emergence to avoid consistent conflicts, and accelerating norm emergence to minimize the duration of the no-norm period. By employing principles of common ground and fairness, the framework helps guide strategy adjustments when conflicts occur. Theoretical and experimental analyses demonstrate the effectiveness and scalability of the proposed framework. A vehicle movement simulator is also developed to vividly demonstrate the norm emergence process.

The contributions of this paper can be summarized as follows:

- This paper presents a novel framework that specifically addresses the unique challenges of norm emergence in IIoT environments. The framework integrates the fusion of agent beliefs about norms with local data, enabling agents to make informed decisions despite the lack of global visibility. This integration helps resolve conflicts, facilitate the emergence of norms, and accelerate the process.
- The proposed framework is both scalable and effective, as demonstrated by comprehensive theoretical analyses and experimental validation, including the development of a vehicle movement simulator to vividly showcase the norm emergence process.

The rest of this paper is organized as follows. Section 2 provides preliminaries. Section 3 describes the proposed decision-making framework. Section 4 presents the theoretical analysis. Section 5 details the experiments. Section 6 discusses related work, and Section 7 concludes the paper.

## 2. Preliminaries

In this section, we offer some background of norm emergence and also the basic definitions for conflict-blocking interactions.

### 2.1. Coordination Games and Norms

Norm emergence is extensively studied from a game-theoretic perspective, where norms might emerge through agent interactions [5]. An interaction is framed by a coordination game [6]. Table 1a,b show two examples of two-player two-strategy coordination games, indicating two road scenarios that vehicles might encounter. Table 1a shows norms where all agents either give way to the left or right at intersections. Similarly, Table 1b shows the norms where all agents move on either the right or left side of the road.

Based on coordination games, norms are defined as follows.

**Definition** **1** (Norm [7]). *Let N be an agent set, g be a coordination game, $s_{i}^{r}$ and $s_{i}^{c}$ be the row strategy and column strategy of an agent i. A norm indicates a situation where the strategy pairs $(s_{i}^{r},s_{j}^{c}),\forall i,j\in N$ are the same pure-strategy Nash equilibrium of g.*

### 2.2. Networked Multi-Agent Systems (MASs) and Agent Interactions

We use an MAS to model a set of agents in an IIoT environment. A networked MAS can be represented by a graph G=(V,E) where *V* is a set of vertices and each vertex represents an agent, E⊆V×V is a set of undirected edges and each edge connects two agents. An agent’s neighbors are those connected with the agent.

### 2.3. Basic Definitions for Conflict-Blocking Interactions

In MASs, particularly those modeling IIoT environments, interactions between agents are crucial. These interactions can lead to coordination or conflict, depending on the strategies chosen by the agents involved. In this section, we introduce key definitions that form the foundation for understanding conflict-blocking interactions, a specific type of interaction where conflicts can impede progress.

We begin by defining the concept of an interaction itself, then move on to explain what constitutes a conflict within these interactions. Following this, we introduce the notion of interaction progress states which helps us track whether an interaction is completed or remains unfinished. Finally, we distinguish between conflict-non-blocking and conflict-blocking interactions, highlighting the critical difference in how conflicts affect the progress of these interactions.

These definitions are essential for understanding the dynamics of agent interactions in complex systems, particularly when dealing with scenarios where conflicts can potentially disrupt the normal functioning of the system. By clearly defining these concepts, we lay the groundwork for developing our framework.

**Definition** **2** (Interaction). *An interaction is defined as $I=\langle g,i,j\rangle$, where g is a coordination game, i and j are the row player and column player of g, respectively. Interaction I represents a situation where players i and j engage in game g where i takes row strategy $s_{i}^{r}$ and j takes column strategy $s_{j}^{c}$.*

**Definition** **3** (Conflict). *Given $I=\langle g,i,j\rangle$, a conflict is defined as a situation where $\left(s_{i}^{r},s_{j}^{c}\right)$ is not a pure-strategy Nash equilibrium of g.*

Based on Definition 3, we can say a strategy pair is either *conflicting* or *conflict-free*.

**Definition** **4** (Interaction Progress State). *Given I, the progress state of I is defined as progress(I)∈{unfinished,finished}.*

**Definition** **5** (Conflict-Non-Blocking Interaction). *Given $I=\langle g,i,j\rangle$ and progress(I) = unfinished, I is a conflict-non-blocking interaction when i,j execute either conflicting or conflict-free strategies, progress(I) changes to finished.*

**Definition** **6** (Conflict-Blocking Interaction). *Given Interaction $I=\langle g,i,j\rangle$ with progress(I)=unfinished, I is a conflict-blocking interaction when i,j execute conflicting strategies, progress(I) remains unfinished; and when i,j execute conflict-free strategies, progress(I) changes to finished.*

The “conflict-blocking” feature stimulates the development of the proposed framework. Some abbreviations and symbols used across sections are summarized in Table 2.

## 3. Decision-Making Framework for Conflict-Blocking Interactions

This section describes the proposed framework. An overview is given first followed by the details.

### 3.1. Overview

**Figure 1:**
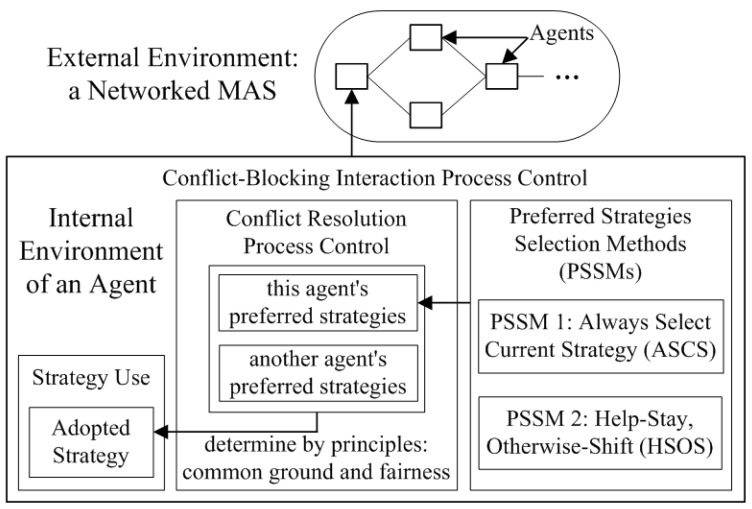
Overview of the proposed decision-making framework.

Figure 1 offers an overview of the proposed framework, which includes four components.

#### 3.1.1. Strategy Usage

This component ensures that an agent uses a *PSNE strategy* to interact with another agent, which provides a basis to fulfill the *Desire for Norm Emergence*. The definition of PSNE strategies, a property of them, and the way of using them are described in Section 3.2.

#### 3.1.2. Conflict Resolution

This component controls a conflict resolution process performed by agents when a conflict occurs so as to fulfill the *Desire for Conflict Resolution*. In the process, the concepts of *preferred strategies*, *common ground*, and *fairness* naturally exist. The conflict resolution process and the involved concepts are described in Section 3.3.

#### 3.1.3. Preferred Strategy Selection Method

The method is used by an agent to select its preferred strategies in a conflict resolution process. We develop two methods to fulfill the *Desire for Norm Emergence* and *Desire for Accelerating Norm Emergence*, respectively. The two methods are described in Section 3.4.

#### 3.1.4. Conflict-Blocking Interaction

This component controls a conflict-blocking interaction process where the above three components are used. The process is given in Section 3.5.

### 3.2. PSNE Strategies: Definition, Property and Usage

#### 3.2.1. Definition of PSNE Strategies

**Definition** **7** (PSNE Strategy). *Given an agent i and a coordination game g, i’s row and column strategies pair $\left(s_{i}^{r},s_{i}^{c}\right)$ is a PSNE Strategy when $\left(s_{i}^{r},s_{i}^{c}\right)$ is a pure-strategy Nash equilibrium of g.*

From an agent’s perspective, adopting a PSNE strategy pair is the necessary condition of norm emergence. With Definition 7, we can say that a norm emerges when all agents adopt the same PSNE strategy.

#### 3.2.2. Property and Usage of PSNE Strategies

For a coordination game, a strategy pair which is a PSNE can also denote a PSNE strategy (the concepts of “PSNE” and “PSNE Strategy” have a slight difference in the scope of usage. “PSNE” implies a multi-agent circumstance, e.g., an agent’s row strategy and another agent’s column strategy are a PSNE. “PSNE Strategy” is defined in a single-agent circumstance, i.e., an agent’s row and column strategies are a PSNE). Hence, PSNEs and PSNE strategies share the same properties. A property used in this paper is the following.

**Property** **1.** *For agent i,j’s PSNE strategies $s_{i}=\left(s_{i}^{r},s_{i}^{c}\right)$, $s_{j}=\left(s_{j}^{r},s_{j}^{c}\right)$, either $s_{i}^{r}=s_{j}^{r}$ and $s_{i}^{c}=s_{j}^{c}$, denoted as $s_{i}=s_{j}$, or $s_{i}^{r}\neq s_{j}^{r}$ and $s_{i}^{c}\neq s_{j}^{c}$, denoted as $s_{i}\neq s_{j}$, holds [6].*

This property indicates that two agents’ PSNE strategies are either at the same row and column in a coordination game’s payoff matrix or at different rows and columns. Based on this property, we propose a proposition for ease of describing how an agent uses its PSNE strategy.

**Proposition** **1.** *For agents i,j’s PSNE strategies $s_{i}=\left(s_{i}^{r},s_{i}^{c}\right)$, $s_{j}=\left(s_{j}^{r},s_{j}^{c}\right)$, $\left(s_{i}^{r},s_{j}^{c}\right)$ is conflicting $\Leftrightarrow s_{i}\neq s_{j}$, $\left(s_{i}^{r},s_{j}^{c}\right)$ is conflict-free $\Leftrightarrow s_{i}=s_{j}$.*

**Proof of Proposition** **1.** When $\left(s_{i}^{r},s_{j}^{c}\right)$ is conflicting, we assume $s_{i}=s_{j}$ indicates that $\left(s_{i}^{r},s_{j}^{c}\right)$ is conflict-free according to Property 1 and Definition 3, which results in a contradiction. Hence, $\left(s_{i}^{r},s_{j}^{c}\right)$ is conflicting $\Rightarrow s_{i}\neq s_{j}$, and the other implications can also be proven by contradictions.    □

When a row player agent *i* and a column player agent *j* interact with each other, *i* uses $s_{i}^{r}$ and *j* uses $s_{j}^{c}$. *i* and *j* know that $s_{i}\neq s_{j}$/$s_{i}=s_{j}$ if $\left(s_{i}^{r},s_{j}^{c}\right)$ is conflicting/conflict-free according to Proposition 1. Hence, for agent *i*, using $s_{i}$ can be seen as comparing $s_{i}$ with another $s_{j}$. $s_{i}\neq s_{j}$ indicates a conflict and $s_{i}=s_{j}$ indicates no conflict.

Hereafter, for simplified descriptions, we use “strategy” to denote “PSNE strategy”, and use “*s*” (non-bold) to denote “s” (bold).

### 3.3. Conflict Resolution Process

A conflict resolution process is performed by interacting agents when a conflict occurs. A conflict means that the agents adopt different strategies according to Proposition 1. To resolve the conflict, the agents need to adopt the same strategy. For the question of which strategy to adopt, an agent prefers some particular strategies, named preferred strategies. An example preferred strategy is the agent’s current strategy, which shows the agent’s incentive of avoiding strategy-changing costs. This example indicates that the interacting agents might have different preferred strategies. If so, the agents need to make an agreement about which preferred strategy to adopt. To make such agreements, the agents can use two principles: common ground and fairness. Both can be widely used in human societies.

Algorithm 1 offers the conflict resolution process. The process is triggered when a conflict is detected. An agent can detect a conflict by using, e.g., collision detection devices [8] when two agents approach each other on the same lane. The steps of the process can be described as follows:

**Step 1:** The algorithm takes as input two agents *i* and *j* that are performing a conflict-blocking interaction with conflicting strategies.

**Step 2:** Agent *i* selects its preferred strategies using a specified selection method.

**Step 3:** Agent *i* obtains agent *j*’s preferred strategies.

**Step 4:** The algorithm checks if there is any overlap between the preferred strategies of agents *i* and *j*.

**Step 5:** If there is an overlap, a conflict-free strategy is randomly chosen from the intersection of their preferred strategies.

**Step 6:** If there is no overlap, a conflict-free strategy is randomly chosen from the union of their preferred strategies.

**Step 7:** The algorithm returns the chosen conflict-free strategy.
**Algorithm 1:** An agent *i*’s conflict resolution process
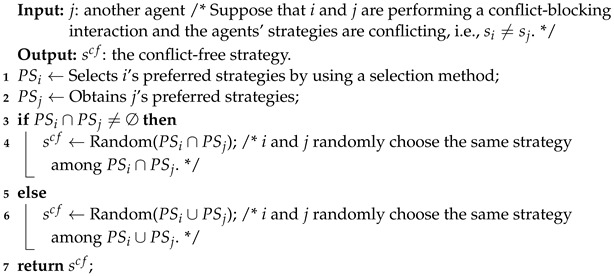


The random choices in Line 4 and Line 6 have different purposes. In Line 4, the agents find some common ground, and the random choice is to choose specific common ground. In Line 6, the agents do not find common ground, and the random choice is to achieve fairness.

### 3.4. Preferred Strategy Selection Methods

The agents in a conflict resolution process select some preferred strategies. These preferred strategies influence the strategy adopted by the agents after conflict resolution. As the strategies adopted by all agents determine whether a norm has emerged, the selection of preferred strategies influences the results of norm emergence, i.e., whether a norm could emerge as well as the time spent for norm emergence.

Based on the *Desire for Norm Emergence* and the *Desire for Accelerating Norm Emergence*, an agent selects the preferred strategies that help fulfill these two desires. To this end, we develop two selection methods: Always Select Current Strategy (ASCS) and Help-Stay, Otherwise-Shift (HSOS).

*Method 1: ASCS*. An agent selects its current strategy as the preferred strategy in all interactions.

ASCS could have wide applicability because (1) it requires minimal agent ability, i.e., selecting current strategy does not even require any calculation; and (2) selecting current strategy is in accordance with an agent’s incentive of avoiding strategy-changing cost. Applying ASCS in Algorithm 1 guarantees norm emergence (see Theorem 1). The time of norm emergence of ASCS is usually longer than the theoretically minimum time (see Remark 1), which encourages the development of HSOS to facilitate faster norm emergence than that of ASCS. Due to the wide applicability and the space for accelerating norm emergence, we regard that ASCS fulfills the *Desire for Norm Emergence* but not the *Desire for Accelerating Norm Emergence*. The average time of norm emergence of ASCS can be seen as a baseline to measure HSOS’s degree of fulfilling the *Desire for Accelerating Norm Emergence*.

*Method 2: HSOS*. At first, an agent *selects* its *local majority strategy* (SLMS) as the preferred strategy. When SLMS is in use, the agent periodically checks whether SLMS helps norm emergence. If it helps, the agent stays using SLMS. Otherwise, the agent begins to *select* its *current strategy* (SCS) as the preferred strategy in subsequent interactions, i.e., the agent shifts from SLMS to SCS.

We first describe the aim of SLMS. We let *majority strategy* (MS) be the strategy mostly adopted in an MAS, *local majority strategy* be the strategy mostly adopted in an agent’s *local area* (the agent and its neighbors), $R_{s}$ be the ṟate of selecting MS as a preferred strategy, and $R_{a}$ be the ṟate of adopting MS for conflict resolution. SLMS aims at promoting higher $R_{s}$ with two thoughts: (1) higher $R_{s}$ results in higher $R_{a}$, and (2) higher $R_{a}$ results in faster norm emergence. For the first thought, when agents select MS as a preferred strategy more often, MS has a higher chance to be chosen as the conflict-free strategy for conflict resolution either by finding common ground or by fair choice. For the second thought, when agents adopt MS for conflict resolution more often, the proportion of agents who adopt MS increases to 100% more quickly, which means faster norm emergence.

The maximum $R_{s}$ and $R_{a}$ are one. $R_{s}=R_{a}=1$ means that agents select and adopt MS in every conflict resolution process, which results in the fastest norm emergence. However, as MS is global information, an agent usually does not know which strategy is MS. In this situation, the agent has to make guesses of MS, which is achieved by SLMS. To utilize possible correct guesses and also avoid failure of norm emergence due to wrong guesses, the idea is to “use SLMS if it helps norm emergence, and shift to SCS otherwise”. To check if SLMS helps, we propose the following definitions.

**Definition** **8** (Local Conformity). *For agent i, its local conformity $lc_{i}=\max_{s}\left(c_{i}\left(s\right)/n_{i}\right)$ where $c_{i}\left(s\right)$ is the number of agents who adopt s in i’s local area (i and its neighbors), and $n_{i}$ is the number of agents in i’s local area.*

The maximum of lc is one. lc=1 means that an agent and its neighbors adopt the same strategy. From an agent’s perspective, lc=1 is the necessary condition of norm emergence. Hence, increasing lc and maintaining it at one can be seen as helping norm emergence. Based on lc, Algorithm 2 shows the decision process of whether to shift from SLMS to SCS. The process is as follows:

**Step 1:** The algorithm takes as input a help-checking interval τ.

**Step 2:** The algorithm checks whether SLMS is currently in use and whether SLMS has been used for τ consecutive conflict resolution processes.

**Step 3:** If the condition in Step 2 is met, the algorithm retrieves the old local conformity value and calculates a new local conformity value.

**Step 4:** The algorithm checks whether the new local conformity value is greater than the old one or the new one has reached one.

**Step 5:** If the condition in Step 4 is met, the agent continues using SLMS and updates the old local conformity value.

**Step 6:** If the condition in Step 4 is not met, the agent shifts from SLMS to SCS.
**Algorithm 2:** An agent *i*’s decision process on whether to shift from SLMS to SCS
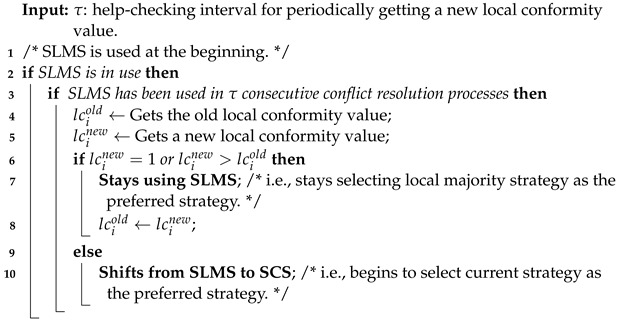


HSOS requires an agent to observe its neighbors when deciding which strategy to select. This neighborhood observation ability is not a strong requirement, and it has been widely used in the literature. Applying HSOS in Algorithm 1 guarantees norm emergence (see Theorem 4), and HSOS facilitates faster norm emergence than that of ASCS with varying degrees in various settings (see Section 5).

### 3.5. Conflict-Blocking Interaction Process

In a conflict-blocking interaction, an agent first checks whether a conflict occurs. If so, the agent calls Algorithm 1 for conflict resolution and then adopts a conflict-free strategy. Next, if HSOS is applied by the agent, Algorithm 2 is called to decide whether to shift from SLMS to SCS. Lastly, the agent executes the conflict-free strategy to finish the interaction.

## 4. Theoretical Analysis

In this section, we offer some theoretical analysis of the proposed framework, including the guarantee and average time of norm emergence, etc. The time spent for a norm to emerge is measured by the *number of conflict resolution processes* that have been performed by all agents before norm emergence. This number is used because a conflict resolution process can be seen as a period where agents make efforts toward norm emergence. Hereafter, the term “time” is used interchangeably with “average time of norm emergence”.

The analysis is conducted from three perspectives:

(1) *Analysis of Applying ASCS in Algorithm 1:* Theorem 1 shows the guarantee of norm emergence. Lemma 1 shows the average time of norm emergence in the two-strategy condition. Theorem 2 shows the maximum time based on Lemma 1. The influences of some typical societal factors on the time are discussed in Factors 1–4. Theorem 3 offers the probability that agents adopt a particular strategy after norm emergence.

(2) *Analysis of Applying HSOS in Algorithm 1:* Theorem 4 shows the guarantee of norm emergence.

(3) *Analysis of Heterogeneous MASs:* Agents who apply ASCS and HSOS could co-exist when some agents lack the observation ability to apply HSOS. In heterogeneous MASs, Corollary 1 deduced from Theorem 4 shows the guarantee of norm emergence.

### 4.1. Analysis of Applying ASCS in Algorithm 1

**Theorem** **1.** *For n agents who adopt m strategies and apply ASCS in Algorithm 1, norm emergence is guaranteed.*

**Proof of Theorem** **1.** We let CR be a conflict resolution process, CR*^s^* be a CR in which an agent’s current strategy is *s*, $E^{\delta }$ be an event that “δ number of consecutive CR*^s^* has been performed” where δ is a fixed number, $c_{s}$ be the number of agents who adopt *s*, ψ and ¬ψ be the situations of $\left(c_{s}=n\right)\vee \left(c_{s}=0\right)$ and $0<c_{s}<n$, respectively, $P(\psi^{\left(1\right)})$ be the probability of being in ψ after $E^{\delta }$ happens once. We can set $E^{\delta }$ to be $E^{n-1}$ so that $P\left(\psi^{\left(1\right)}|\neg \psi^{\left(\omega \right)}\right)\geq {\left(1/2\right)}^{n-1}>0,\omega \in N$. Note that ${\left(1/2\right)}^{n-1}$ indicates a process where $c_{s}=1$ in $\neg \psi^{\left(\omega \right)}$, and the agents in $E^{n-1}$ all adopt *s* (where 1/2 is due to the random choice of the conflict-free strategy). Then, we have $P\left(\neg \psi^{\left(\omega +1\right)}\right)=P\left(\neg \psi^{\left(\omega \right)}\right)P\left(\neg \psi^{\left(1\right)}|\neg \psi^{\left(\omega \right)}\right)$ where $P\left(\neg \psi^{\left(1\right)}|\neg \psi^{\left(\omega \right)}\right)=1-P\left(\psi^{\left(1\right)}|\neg \psi^{\left(\omega \right)}\right)<1$, which indicates that event $E^{n-1}$ results in a contraction on P(¬ψ) with a fixed point zero. Hence, when ω→∞, $P(\neg \psi^{\left(\omega \right)})\to 0$, and $P(\psi^{\left(\omega \right)})\to 1$. This means that when agents continuously perform CR*^s^*, *s* is (i) either adopted by all agents or (ii) not adopted by any agents. For each of the *m* strategies, (i) or (ii) happens, which means that there is only one strategy adopted by all agents, i.e., a norm emerges. □

The proof of Theorem 1 uses probability theory to show that as agents repeatedly engage in conflict resolution processes, the system eventually reaches a state where all agents adopt the same strategy, leading to the emergence of a norm.

**Lemma** **1.** *For n agents who adopt two strategies and apply ASCS in Algorithm 1, when a strategy is adopted by c agents, the average time of norm emergence $t_{c}=-c^{2}+cn$.*

**Proof of Lemma** **1.** After a CR (CR and CR*^s^* defined in previous proof are used throughout this section), *c* has an equal probability of increasing or decreasing by one. Hence, the probability that *c* increases to *n* or decreases to zero (i.e, the probability of norm emergence) exactly after δ number of CR, denoted as $p_{c}^{\left(\delta \right)}$, is well defined. Then, $t_{c}$ is also well defined ($t_{c}=\sum_{\delta =0}^{\infty }p_{c}^{\left(\delta \right)}\delta$). Instead of computing $p_{c}^{\left(\delta \right)}$, we can compute $t_{c}$ directly using equations

(1) $$\left\{\begin{array}{c} t_{1}=1+t_{0}/2+t_{2}/2\\ \vdots \\ t_{c}=1+t_{c-1}/2+t_{c+1}/2\\ \vdots \\ t_{n-1}=1+t_{n-2}/2+t_{n}/2, \end{array}\right.$$

where $t_{c}=1+t_{c-1}/2+t_{c+1}/2$ means that after 1 CR, all agents have 1/2 probability of still requiring the time of $t_{c-1}$ or $t_{c+1}$ for norm emergence. Also, we have $t_{0}=0$ and $t_{n}=0$ as (c=0)∨(c=n) means that a norm has already emerged. Then, we can verify that the general term of Equation (1) is $-c^{2}+cn$ by (a) substituting it for $t_{c-1}$ and $t_{c+1}$ in $1+t_{c-1}/2+t_{c+1}/2$, which results in $1+\left(-{\left(c-1\right)}^{2}+\left(c-1\right)n\right)/2+\left(-{\left(c+1\right)}^{2}+\left(c+1\right)n\right)/2=-c^{2}+cn$, and (b) verifying that $t_{0}=-0^{2}+0n=0$ and $t_{n}=-n^{2}+nn=0$. □

Lemma 1 quantifies the average time required for a norm to emerge when agents can adopt one of two strategies. The time is determined by calculating the expected number of conflict resolution processes needed for all agents to converge on a single strategy. The time depends on the number of agents initially adopting each strategy.

**Theorem** **2.** *For n agents who adopt m strategies and apply ASCS in Algorithm 1, the maximum average time of norm emergence is $\left(m-1\right)n^{2}/\left(2m\right)$, which is achieved when each strategy is adopted by the same number (n/m) of agents.*

**Proof of Theorem** **2.** We let $t_{\vec{c}}$ be the average time of norm emergence where $\vec{c}=\left\{c_{s^{1}},c_{s^{2}},\ldots ,c_{s^{m}}\right\}$ indicates the number of agents that adopt each of the *m* strategies. $t_{\vec{c}}$ can be calculated by

(2) $$t_{\vec{c}}=\frac{1}{2}\sum_{k=1}^{m}\left(-c_{s^{k}}^{2}+c_{s^{k}}n\right),$$

where $-c_{s^{k}}^{2}+c_{s^{k}}n$ is the number of CRs with respect to $s^{k}$ after norm emergence based on Lemma 1, and the division by two is needed because each CR is counted twice with respect to two strategies. Then, we can set $c_{s^{m}}=n-\sum_{i=1}^{m-1}c_{s^{i}}$ in Equation (2) and find its maximum using partial derivatives, e.g., $\partial t_{\vec{c}}/\partial c_{s^{k}}=-2c_{s^{k}}-\sum_{i=1,i\neq k}^{m-1}c_{s^{i}}+n=0$. There are m−1 equations with m−1 unknowns, and the solutions are $c_{s^{i}}=n/m,i\in \left\{1,\ldots ,m-1\right\}$. Substituting the solutions into Equation (2) results in $\left(m-1\right)n^{2}/\left(2m\right)$. □

Equation (2) is derived from Lemma 1 by extending the result for two strategies to a general case with *m* strategies. The key steps involve summing the average time contributions from each strategy while accounting for the fact that each CR is counted twice in the two-strategy scenario. Theorem 2 provides an upper bound on the average time for norm emergence when agents adopt multiple strategies and use ASCS for conflict resolution. The maximum time occurs when the strategies are evenly distributed among the agents.

**Remark** **1.** *The time of norm emergence of ASCS is usually longer than the theoretically minimum time of norm emergence. We let $l_{\tilde{s}}=\min\left\{n-c_{s^{1}},\ldots ,n-c_{s^{m}}\right\}$ and $\tilde{s}$ be the corresponding strategy. $l_{\tilde{s}}$ indicates the theoretically minimum time of norm emergence, i.e., the least number of CRs that need to be performed for norm emergence. The probability of norm emergence exactly after $l_{\tilde{s}}$ number of CRs is ${\left(1/2\right)}^{l_{\tilde{s}}}$, which requires that (1) every CR is between an agent who adopts $\tilde{s}$ and another agent who does not adopt $\tilde{s}$, and (2) $\tilde{s}$ is adopted after every CR. Both requirements can be very strong and are rarely satisfied.*

The influences of typical societal factors on the average time of norm emergence are discussed as follows.

**Factor** **1** (Network Structure). *The proof for Lemma 1 and Theorem 2 implicitly indicates that network structure has no influence on the time because the increase or decrease in $c_{s}$ by one after CR^s^ has no relationship to the pair of agents who perform CR^s^.*

**Factor** **2** (Number of Strategies, *m*). *m has a bounded influence on time. This can be shown by writing the maximum time in Theorem 2 as $\left(1/2-1/\left(2m\right)\right)n^{2}$. The theoretically maximum m is n and the theoretically maximum time is (n−1)n/2.*

**Factor** **3** (Number of Agents, *n*). *n has a quadratic growth influence on the time, which can be shown by examining the maximum and minimum time. For the maximum time, it is represented in Theorem 2, which shows a quadratic growth influence. For the minimum time, it can be represented by −1+n based on the condition of c=1 in Lemma 1. −1+n shows a linear growth influence. However, meeting the c=1 condition requires that a strategy is always adopted by one (fixed number) agent across different MASs with different n. The fixed c condition is very strong. A more reasonable condition can be that c is in proportion to n. For example, c=n/2 can indicate that agents’ strategies are randomly initialized. Following this, we can use $n^{2}/4$ to represent the minimum time, and $n^{2}/4$ shows a quadratic growth influence.*

**Factor** **4** (Proportion of Agents who Adopt MS, μ). *μ has a quadratic drop influence on the time. We first examine the maximum time. We let $\hat{s}$ be MS and A/¬A be the agents who adopt/do not adopt $\hat{s}$ initially. We can write Equation (2) as*

(3) $$t_{\vec{c}}=-c_{\hat{s}}^{2}+c_{\hat{s}}n+\frac{1}{2}\sum_{k=1,s^{k}\neq \hat{s}}^{m}\left(-c_{s^{k}}^{2}+c_{s^{k}}\left(n-c_{\hat{s}}\right)\right),$$

*where $-c_{\hat{s}}^{2}+c_{\hat{s}}n$ is the number of CRs performed between an agent in A and another agent in ¬A; the rest of Equation (3) is the number of CRs performed between agents in ¬A, and this number’s maximum value can be calculated using Theorem 2. Then, Equation (3) can be written as*

(4) $$\begin{array}{cc} t_{\vec{c}} & =-{\left(\mu n\right)}^{2}+\mu n^{2}+\frac{\left(m-1-1\right){\left(n-\mu n\right)}^{2}}{2(m-1)}\\ \; & =\frac{\left(-m\mu^{2}+2\mu +m-2\right)n^{2}}{2(m-1)}, \end{array}$$

*where 1/m≤μ≤1. When μ=1/m, Equation (4) reaches its maximum value $\left(m-1\right)n^{2}/\left(2m\right)$, which is in line with Theorem 2. With larger μ, Equation (4) shows a quadratic drop. For the minimum time, similar to the previous factor, we use Lemma 1 and consider that c is in proportion to n. We can set c=μn, and the minimum time is $\left(-\mu^{2}+\mu \right)n^{2}$ where 1/2≤μ≤1, which also shows that μ has a quadratic drop influence.*

**Theorem** **3.** *For n agents who adopt m strategies and apply ASCS in Algorithm 1, when the strategy s is adopted by $c_{s}$ agents, the probability that all agents adopt s after norm emergence is $p_{c_{s}}^{s}=c_{s}/n$.*

**Proof of Theorem** **3.** Following the proof for Lemma 1, the probability that all agents adopt *s* exactly after δ number of CR*^s^*, denoted as $p_{c_{s}}^{s(\delta )}$, is well defined. Hence, $p_{c_{s}}^{s}$ is also well defined ($p_{c_{s}}^{s}=\sum_{\delta =0}^{\infty }p_{c_{s}}^{s(\delta )}$). We can compute $p_{c_{s}}^{s}$ directly using equations

(5) $$\left\{\begin{array}{c} p_{1}^{s}=p_{0}^{s}/2+p_{2}^{s}/2\\ \vdots \\ p_{c_{s}}^{s}=p_{c_{s}-1}^{s}/2+p_{c_{s}+1}^{s}/2\\ \vdots \\ p_{n-1}^{s}=p_{n-2}^{s}/2+p_{n}^{s}/2, \end{array}\right.$$

where $p_{c_{s}}^{s}=p_{c_{s}-1}^{s}/2+p_{c_{s}+1}^{s}/2$ means that after a CR, all agents have a half chance of still having $p_{c_{s}-1}^{s}$ or $p_{c_{s}+1}^{s}$ probability of adopting *s* after norm emergence. Also, we have $p_{0}^{s}=0$ and $p_{n}^{s}=1$. Then, we can verify that the general term of Equation (5) is $c_{s}/n$ by checking that $p_{c_{s}}^{s}=\left(c_{s}-1\right)/\left(2n\right)+\left(c_{s}+1\right)/\left(2n\right)=c_{s}/n$, $p_{0}^{s}=0/c=0$, and $p_{n}^{s}=n/n=1$. □

Theorem 3 calculates the probability that all agents adopt a particular strategy after norm emergence. The probability is directly proportional to the number of agents initially adopting that strategy. The proof is based on the recurrence relations similar to those used in Lemma 1.

### 4.2. Analysis of Applying HSOS in Algorithm 1

**Theorem** **4.** *For n agents who apply HSOS in Algorithm 1, norm emergence is guaranteed.*

**Proof of Theorem** **4.** When agents apply HSOS in Algorithm 1 for sufficiently long time, one of the three results happens:

*Result 1:* No agents shift from SLMS to SCS. In this result, all agents’ lc values are increased to one. An agent’s lc=1 means that the agent and its neighbors adopt the same strategy. Hence, all agents’ lc=1 mean that they adopt the same strategy, i.e., a norm emerges.

*Result 2:* All agents shift from SLMS to SCS. This result is equivalent to that all agents apply ASCS, which guarantees norm emergence according to Theorem 1.

*Result 3:* Some of the agents shift from SLMS to SCS. In this result, we can regard that all agents are grouped into several regions where a region has agents who have shifted or not. We let $R_{SLMS}$ and $R_{SCS}$ be two connected regions. The agents in $R_{SLMS}$ adopt the same strategy, denoted as $s(R_{SLMS})$, according to Result 1. Also, the agents in $R_{SCS}$ adopt the same strategy, denoted as $s(R_{SCS})$, according to Theorem 1. This theorem holds for $R_{SCS}$ because although some agents in $R_{SCS}$ are connected with $R_{SLMS}$, results (i) and (ii) in the proof still happen. Then, we have $s\left(R_{SLMS}\right)=s\left(R_{SCS}\right)$ because if $s\left(R_{SLMS}\right)\neq s\left(R_{SCS}\right)$, the agents in $R_{SLMS}$ who are connected with $R_{SCS}$ cannot increase their lc to one and shift. Hence, agents in every pair of connected regions adopt the same strategy, and a norm emerges.

□

Theorem 4 guarantees that a norm will emerge when agents use HSOS. The proof outlines three possible results depending on whether agents shift from SLMS to SCS or not, and shows that in all cases, agents eventually adopt a single strategy, resulting in norm emergence.

We discussed the influences of Factors 1–4 on the average time of norm emergence of ASCS. These factors could also influence the time of HSOS, which is experimentally studied in Section 5.

### 4.3. Analysis of Heterogeneous MASs

**Corollary** **1.** *For n agents where some of the agents apply HSOS and the other agents apply ASCS in Algorithm 1, norm emergence is guaranteed.*

**Proof of Corollary** **1.** See *Results 2* and *3* in the proof for Theorem 4. □

Corollary 1 extends Theorem 4 to heterogeneous MAS, where some agents use HSOS while others use ASCS. The proof relies on the results described in Theorem 4, ensuring that the system converges to a single strategy. The key difference between norm emergence in homogeneous and heterogeneous systems lies in the dynamics of the conflict resolution processes. In homogeneous MASs, the average time is more predictable and can be calculated with formulas. In contrast, in heterogeneous MASs, the time could be influenced by agents applying HSOS and the factors relating to those agents. In this paper, we study the following societal factor:

**Factor** **5** (Proportion of Agents who Apply HSOS, *h*). A bigger *h* means that more agents have the neighborhood observation ability to apply HSOS.

The influence of *h* on the average time of norm emergence is studied in Section 5.

## 5. Experiments

This section experimentally evaluates the proposed framework. We first offer the settings of six experiments and then show the results and analysis. The vehicle movement simulator is described at the end of this section.

### 5.1. Settings

#### 5.1.1. Experiment Scenarios

The experiments aim to achieve two main goals: (1) determining whether HSOS leads to faster norm emergence compared to ASCS and (2) assessing the impact of Factors 1–5 on the average norm emergence time for HSOS and ASCS.

*Experiment 1* investigates the influence of various network structures on HSOS given its reliance on neighbor interactions.

*Experiment 2* analyzes the effects of HSOS’s three core components: SLMS, the help-checking interval τ, and the transition from SLMS to SCS.

*Experiments 3–6* explore how different values of Factors 2–5 impact the emergence time.

The current approaches for norm emergence are not applied in the experiments because these approaches are based on conflict-non-blocking interactions. If agents apply them, the agents cannot finish conflict-blocking interactions when conflicts occur, and the experiments are interrupted.

#### 5.1.2. Networks and Parameters

In the experiments, conflict-blocking interactions occur simultaneously in an MAS. The network structure of the MAS determines which pairs of agents can interact. We use four types of networks commonly studied in the literature: fully connected (FC), small-world (SW), scale-free (SF), and community (CM) networks.

In FC, any two agents are neighbors.

In SW, each agent has a limited number of neighbors, and the path length between any two agents tends to be short [9]. Examples include social influence networks [10]. We generate SW using the Watts–Strogatz model [9] with parameters *n*, *b*, and ρ, where *n* is the number of agents, *b* is the average number of neighbors, and ρ is the rewiring probability of an edge.

In SF, the number of neighbors *k* follows a power-law distribution $k^{-\gamma }$ [11], similar to citation networks between scientific papers [12]. We use the Barabási–Albert model [13] to generate SF with parameters *n* and λ, where *n* is the number of agents, and λ is the number of edges a new node attaches to existing nodes.

In CM, agents are organized into several communities where intra-community connections are more likely than inter-community connections [14]. Examples include biological networks [15]. We generate CM using the Gaussian random partition generator [16] with parameters *n*, *u*, *v*, and *r*, where *n* is the number of agents, *u* is the number of communities, *v* is the variance in the number of agents per community, and *r* is the ratio of intra-community neighbors to total neighbors. A higher *r* indicates more distinct community structures.

Unless otherwise specified, we use the following default parameters for network generation. We set the number of agents n=100 for all networks. For SW, we set b=4 for a few neighbors and ρ=0.5 for moderate edge randomness [9]. For SF, we set λ=1, resulting in a power-law exponent γ of approximately 2.34 [13]. For CM, we set r=0.95 to signify a strong community structure [17], with u=10 and v=1. Besides network parameters, we set the number of strategies m=2, the proportion of agents adopting MS μ=1/m, the proportion of agents using HSOS h=0/h=1 to indicate that all agents use ASCS/HSOS, and set the HSOS help-checking interval τ=5. Results are averaged over 1000 independent runs.

### 5.2. Results and Analysis

#### 5.2.1. Tests with Various Network Structures

**Figure 2:**
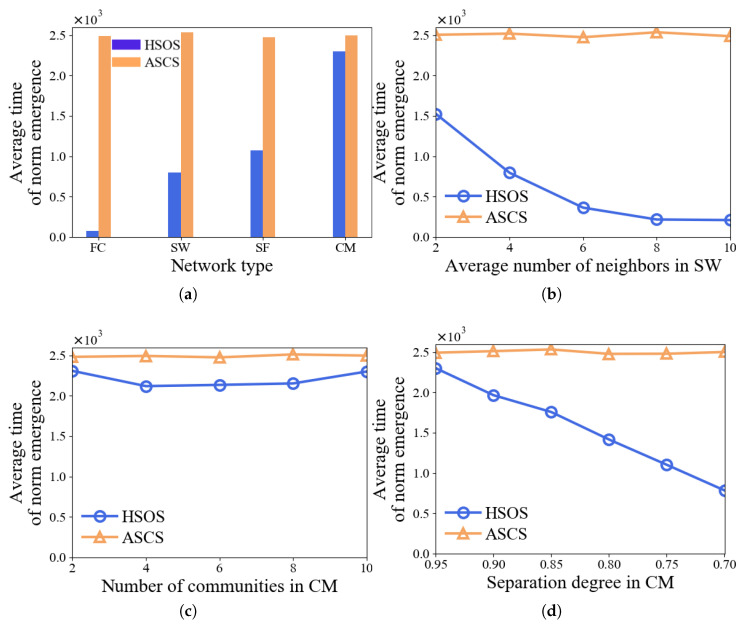
Average time of norm emergence of HSOS and ASCS with various network structures (**a**–**d**).

Figure 2a illustrates the average time for norm emergence using HSOS and ASCS across four different network types. HSOS consistently shows quicker norm emergence compared to ASCS, especially in FC networks, and to a lesser extent in CM networks. Additionally, we alter specific network parameters within SW and CM. Figure 2b demonstrates that in SW networks, increased neighbor connections among agents accelerate norm emergence with HSOS. Figure 2c indicates that the number of communities in CM networks does not significantly affect HSOS norm emergence time. Figure 2d reveals that higher separation degrees in CM networks lead to faster norm emergence with HSOS. These findings collectively indicate that HSOS enables quicker norm emergence than ASCS across various network structures, with the network structure itself significantly impacting HSOS performance. To further analyze these results and gain deeper insights into HSOS functionality, we conduct the next experiment.

#### 5.2.2. Illustrations of How HSOS Works

The illustrations analyze the impacts of HSOS’s three main components: (1) SLMS, (2) the help-checking interval τ, and (3) the transition from SLMS to SCS.

*Effect of SLMS:* SLMS aims to achieve a higher $R_{s}$ compared to ASCS through two hypotheses: (1) a higher $R_{s}$ leads to a higher $R_{a}$, and (2) a higher $R_{a}$ accelerates norm emergence (see Section 3.4).

**Figure 3:**
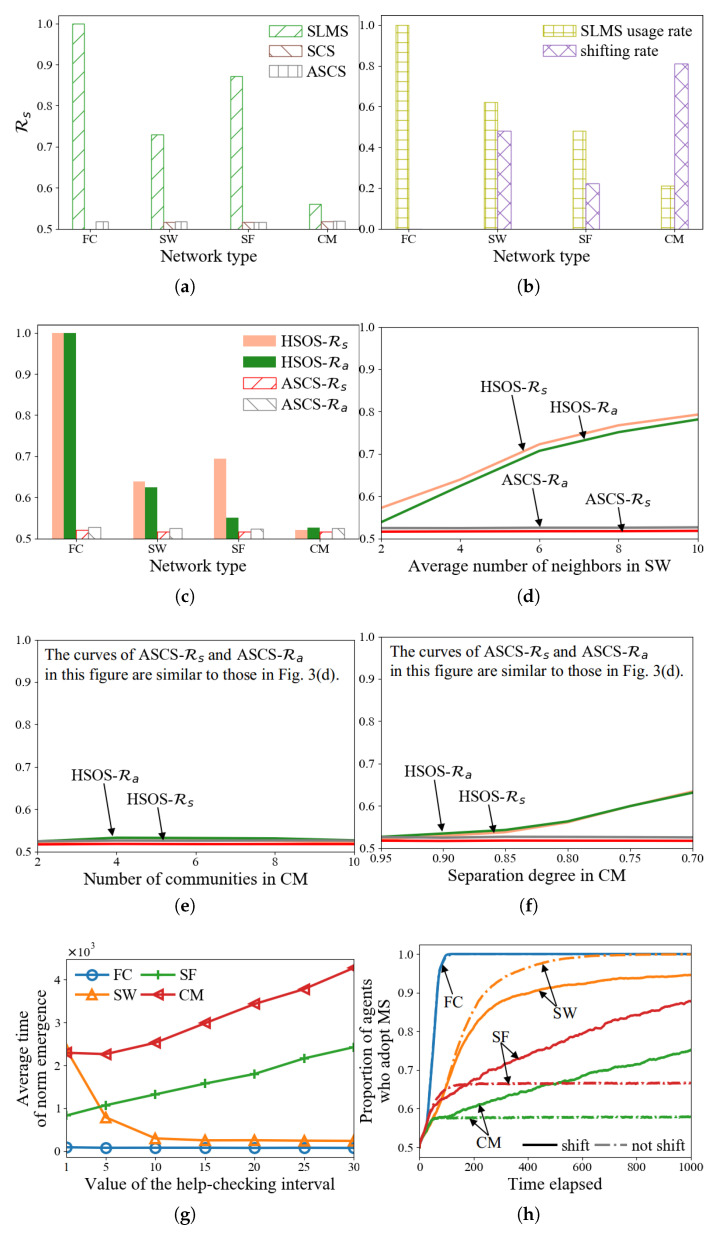
Different kinds of statistics of HSOS and ASCS in various settings (**a**–**h**).

To test these hypotheses, Figure 3a illustrates the $R_{s}$ of SLMS, SCS, and ASCS under the conditions depicted in Figure 2a. SLMS consistently shows a higher $R_{s}$ than ASCS across various network types. Consequently, the average $R_{s}$ of HSOS (which combines SLMS and SCS) surpasses that of ASCS, as shown in Figure 3c (with SLMS usage rates in Figure 3b). Figure 3c–f compare $R_{s}$ and $R_{a}$ of HSOS and ASCS across the scenarios in Figure 2a–d, confirming the strong correlation between $R_{s}$ and $R_{a}$, thus supporting faster norm emergence.

Examining the $R_{s}$ values further, Figure 3a reveals that $R_{s}$ values for ASCS and SCS are nearly identical and slightly above 50% across different networks. This similarity arises because both methods select the current strategy as the preferred one. The minimal variation in $R_{s}$ across networks can be attributed to Factor 1, indicating that network structure does not affect ASCS’s norm emergence time, and thus the related $R_{s}$ statistics. The $R_{s}$ being slightly above 50% can be explained by the selection dynamics: when agent strategies are equally distributed, both selected strategies are MS; otherwise, one of the two strategies is MS in a two-strategy setup, making the average MS selection probability just over 50%.

Figure 3c–f depict the variation of $R_{s}$ in HSOS across networks. In FC, $R_{s}$ approaches 1 due to global visibility and optimal strategy selection via SLMS, resulting in rapid lc increment to 1 and 100% SLMS usage (Figure 3b). Conversely, in CM, $R_{s}$ is low, similar to ASCS. The community structure impedes SLMS’s effectiveness, leading agents within communities to adopt prevalent local strategies, which might differ across communities. Such difference hinders lc increase, causing high shifting rates, low SLMS usage, and reduced $R_{s}$. This trend persists regardless of the number of communities (Figure 3e). A lower separation degree in CM results in higher $R_{s}$ due to increased inter-community interaction (Figure 3f). In SW and SF, $R_{s}$ exceeds that in CM due to more randomized neighbor distribution, making SLMS-selected strategies more representative of the overall population. Higher average neighbor counts in SW enhance $R_{s}$ (Figure 3d). In SF networks, $R_{s}$ is even higher due to many agents having only one neighbor, leading them to be easier to adopt MS during interactions.

*Effect of the help-checking interval τ:* τ defines the frequency of SLMS usage checks for norm emergence assistance. Figure 3g shows the average norm emergence time across various τ values. Larger τ values accelerate norm emergence in FC and SW, but slow it down in SF and CM. The optimal τ varies by network type, which agents typically do not know. In experiments, τ=5 is used empirically, with future work needed to optimize τ based on network characteristics.

*Effect of shifting from SLMS to SCS:* Shifting ensures norm emergence. Figure 3h tracks the proportion of agents adopting MS with and without shifting (where τ=+∞ means no shifting, always using SLMS). Shifting results in all agents eventually adopting MS across all networks. Without shifting, proportions in SF and CM initially rise but stabilize below 1, indicating potential sub-norms. These sub-norms occur due to structural peculiarities like CM’s community structure, leading to stable regional strategy divergence, as discussed in prior studies on norm emergence in complex networks [18,19].

#### 5.2.3. Tests with Various Numbers of Strategies, *m*

In this experiment, we set *m* to values in [2,4,6,8,10] and fix the number of agents *n* at 120. This ensures that each strategy initially has an equal number of agents. The network type also plays a significant role in the results.

**Figure 4:**
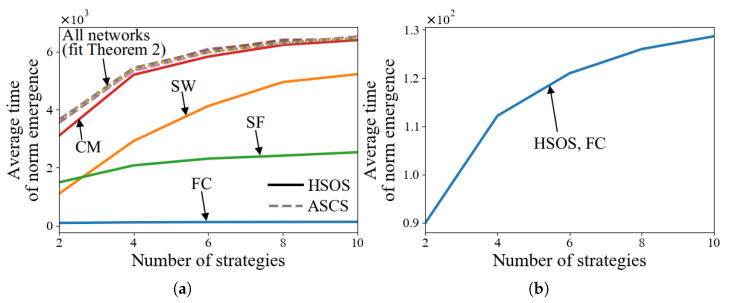
Average time of norm emergence of HSOS and ASCS with various numbers of strategies (**a**,**b**).

Figure 4a shows that HSOS accelerates norm emergence more effectively than ASCS across different *m* values and network types. The acceleration patterns are similar to those in previous experiments: pronounced in FC, minimal in CM, and moderate in SW and SF. The time of ASCS aligns with theoretical predictions (Theorem 2). The influence of *m* on both ASCS and HSOS time is limited, as detailed in Figure 4b for HSOS in FC. For ASCS, this limited influence is discussed in Factor 2.

The results show that regardless of *m*, the reduction rate of existing strategies remains nearly constant. Additionally, Figure 4a reveals that in SF networks, as *m* increases, the time growth rate of HSOS is lower compared to SW and CM networks. This may be due to the frequent strategy changes by one-neighbor agents in SF, leading to a quicker reduction in the number of existing strategies.

#### 5.2.4. Tests with Various Numbers of Agents, *n*

**Figure 5:**
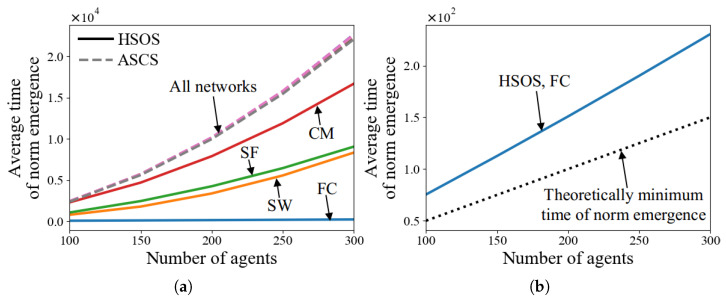
Average time of norm emergence of HSOS and ASCS with various numbers of agents (**a**,**b**).

In this experiment, *n* is set to values in [100,150,200,250,300]. Figure 5a demonstrates that HSOS accelerates norm emergence faster than ASCS across different *n* values and network types. The time for ASCS grows quadratically with *n*, as discussed in Factor 3. For HSOS, a quadratic growth influence on time is also observed in SW, SF, and CM due to the use of SCS. In FC, Figure 5b shows a linear growth influence, indicating significantly faster norm emergence in large *n* settings compared to other networks. However, FC is less common due to its strong requirements in MASs. Future work should aim to achieve linear or near-linear growth influence in more common networks like SW, SF, and CM.

Figure 5b also indicates the theoretically minimum time for norm emergence, showing that when n=100 and each of two strategies is initially adopted by 50 agents, the least number of conflict resolution processes needed is 50. The time in FC is longer than this theoretical minimum because agents may not adopt the same MS after their first conflict resolution processes.

#### 5.2.5. Tests with Various Proportions of Agents Who Adopt MS, μ

**Figure 6:**
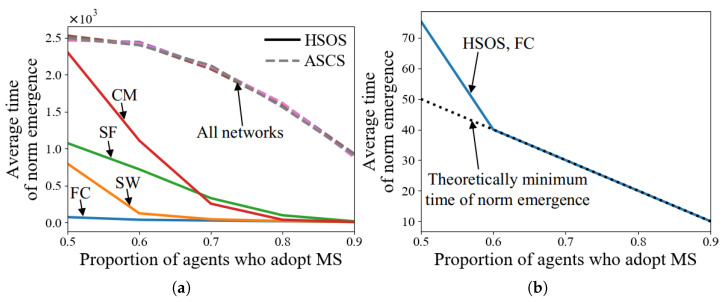
Average time of norm emergence of HSOS and ASCS with various proportions of agents who adopt MS (**a**,**b**).

In this experiment, μ is set in [0.5,0.6,0.7,0.8,0.9]. Figure 6a shows that HSOS facilitates faster norm emergence than ASCS with varying degrees under different μ values across four network types. The time for ASCS decreases quadratically with μ, as discussed in Factor 4. For HSOS, a higher μ results in a quicker decrease in norm emergence time across all networks. This suggests that if a majority of agents could quickly adopt the same strategy, norm emergence would be significantly faster.

**Figure 7:**
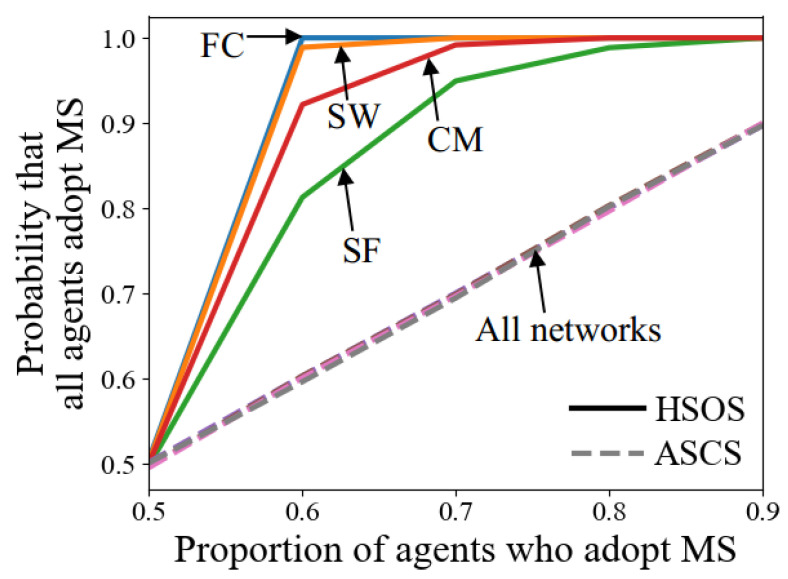
Probability that all agents adopt MS after norm emergence.

Figure 6b shows that when μ>0.5, the time of HSOS in FC reaches the theoretically minimum value, as all agents can observe and adopt the same MS at their first conflict resolution processes. Additionally, μ influences the probability that agents adopt a particular strategy after norm emergence. Figure 7 illustrates this probability in terms of adopting MS, showing that for ASCS, the probability aligns with μ, while for HSOS, the probability increases more quickly with a higher μ due to SLMS usage and finding common ground.

#### 5.2.6. Tests with Various Proportions of Agents Who Apply HSOS, *h*

**Figure 8:**
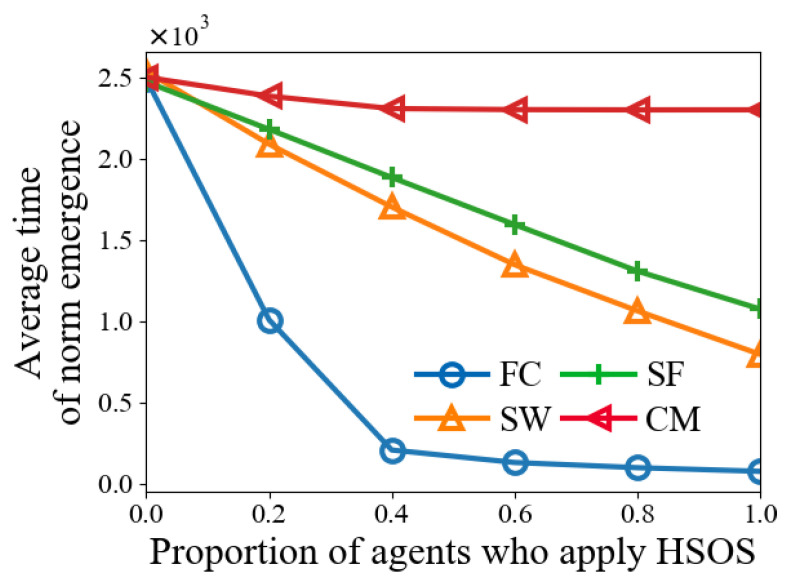
Time results with various proportions of agents who apply HSOS.

In this experiment, *h* is set in [0,0.2,0.4,0.6,0.8,1]. Figure 8 shows that norm emergence is faster with higher *h* values across four network types. More HSOS agents lead to a greater reduction in norm emergence time. The maximum time reduction for a network type can be calculated by the time difference between h=0 and h=1. In SW and SF, the time reduction is almost linear with increasing *h*, while in FC and CM, the time reduces more quickly. These results indicate that even a small proportion of agents applying HSOS can significantly reduce the norm emergence time, suggesting a trade-off between agent ability and time reduction.

#### 5.2.7. Standard Deviation of Time of Norm Emergence, σ

The σ of ASCS is often higher than that of HSOS. For example, in the setting of 100 agents in FC, the σ of ASCS and HSOS are around 2000 and around 10, respectively. In other settings, σ is higher when more HSOS agents shift from SLMS to SCS. The theoretical analysis of σ is left for future work.

### 5.3. Vehicle Movement Simulator

**Figure 9:**
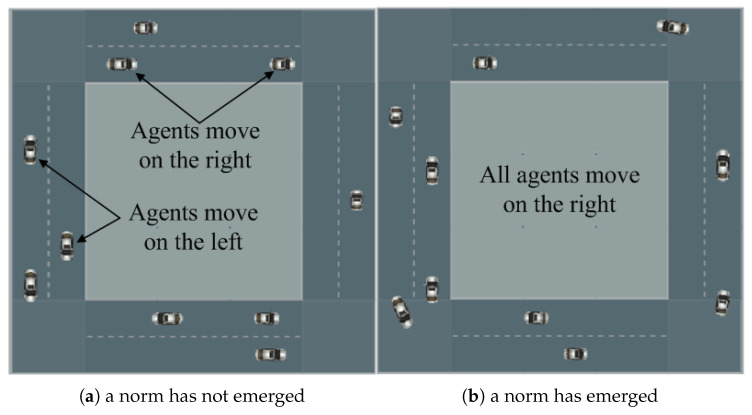
Example road situations.

The primary purpose of the simulator is to vividly illustrate the process of norm emergence through conflict-blocking interactions. Figure 9a,b show two example road situations. A vehicle (agent) can choose to move either on right side or on left side of the road. When two vehicles move toward each other, they perform a passing (conflict-blocking) interaction. The proposed framework is applied by vehicles to perform the interactions (where ASCS is used as the preferred strategy selection method). Vehicles continuously interact with each other and resolve conflicts if necessary, and the road changes from a no-norm situation to a norm situation, as shown in Figure 9a,b, respectively.

The simulator could also be used to represent a dynamic network where the connections between vehicles are continuously changing, and the changes are restricted by the road structure. Two vehicles are regarded as being connected when they are within a predefined spatial distance. Vehicles keep moving on the road, and the connections also keep changing. As indicated by the analysis of Factor 1, the theoretical results like the guarantee and time of norm emergence of the proposed framework still hold in such dynamic network. However, if we try to incorporate popular techniques, such as learning, into the proposed framework, the dynamic network might have a great impact, and we leave this study as future work. Also, the simulator could be updated further for future work, such as to represent more realistic road situations.

### 5.4. Discussions

#### 5.4.1. Key Findings

Both ASCS and HSOS guarantee norm emergence. This rapid convergence is crucial for enhancing the efficiency and reliability of IIoT systems, such as in smart manufacturing where quick consensus among devices reduces downtime.

The SLMS component of HSOS promotes faster norm emergence. The transition from SLMS to SCS guarantees norm emergence across complex network structures, making HSOS a robust choice for dynamic IIoT environments.

The performance of HSOS varies with network topology. While HSOS is effective across various network topologies, its performance is better in networks with higher connectivity and more uniform interactions, such as FC and SW networks. In more unevenly connected networks like CM, additional strategies may be needed to improve HSOS’s effectiveness.

A small proportion of agents using HSOS can significantly reduce norm emergence time across network types. This indicates that in real-world IIoT systems, not all devices need to adopt HSOS for the system to benefit from its advantages, allowing for a gradual or partial implementation in existing systems.

#### 5.4.2. Limitations

The experiments rely on a limited set of predefined network structures, which may not fully represent the diversity of real-world networks.

The experiments use fixed parameters for network generation and other variables. While this approach simplifies analysis, it may overlook the effects of varying these parameters.

The experiments assume that all agents follow the same behavior patterns (e.g., applying HSOS or ASCS). In reality, agents may exhibit more diverse and heterogeneous behaviors, which could affect norm emergence dynamics.

While the vehicle movement simulator provides a controlled environment for illustrating norm emergence, the experiments do not include real-world or more complex simulation scenarios that might better reflect practical applications of the framework.

The conflict-blocking interactions used in the experiments are a simplified model of agent interactions. In more complex or nuanced scenarios, such as those involving multi-step decision processes or learning, the proposed framework might behave differently.

## 6. Related Work

### 6.1. Agent Interactions for Norm Emergence

The current approaches for norm emergence mainly involve learning and spreading interactions. In a learning interaction, an agent adjusted its strategies by using reinforcement learning techniques [20]. In this line of research, Shoham and Tennenholtz [5] defined the notion of social norms in a game-theoretic framework, and investigated the emergence of norms where learning was used to accumulate system-wide information. Airiau et al. [7] proposed a social learning framework based on multi-agent learning, which had been widely used as the basic learning model. Yu et al. [21] proposed a collective learning framework to study the impact of agents’ local collective behaviors on norm emergence. Hao et al. [22] extended the collective learning framework by considering a wider range of interactions where, e.g., only some of Nash equilibria corresponded to norms. Yu et al. [23] studied the influence of organizational structure on norm emergence where each organization contained supervisors who aggregated the learning information. This work was improved by Yang et al. [24] who considered heuristic learning based on supervisors’ instructions. Hu and Leung [17] utilized social learning to coordinate agents’ strategies in communities. Vouros [25] proposed a learning approach which considered multiple tasks, operational constraints, as well as limited interaction and monitoring abilities. Hao et al. [26] proposed a learning approach which considered communication cost in cell systems. Wang et al. [27] used the teacher–student technique to accelerate learning in a large norm space. Leung et al. [28] proposed a model-based learning approach which considered the memory length of agents. In a spreading interaction, an agent transferred its candidates of norms to other agents who then completely or partially copy the received candidates. In this line of research, Delgado [29] used a generalized simple majority rule where an agent probably copied the strategy that was mostly seen in neighbors. Salazar et al. [30] focused on promoting the emergence of high quality norms from a large norm space by utilizing evolutionary algorithms. Franks et al. [31] utilized some influencer agents to improve norm emergence. Hasan et al. [32] used the contextual knowledge of the topology of an MAS to improve norm emergence. All the above approaches, however, are based on conflict-non-blocking interactions and cannot be directly applied to IIoT environments where conflict-blocking interactions are prevalent. Specifically, in a learning interaction, a conflict cannot block an agent’s learning, but instead promotes the agent’s learning by making the agent learn that, e.g., a strategy could cause a conflict. In other words, a learning interaction can be finished no matter whether there is a conflict. Similarly, in a transfer interaction, a conflict does not block the transfer but only influences the transferred information. Overall, this paper deals with the conflict-blocking nature in IIoT environments to enable norm emergence.

### 6.2. Theoretical Analysis of Norm Emergence

Shoham and Tennenholtz [5] and Brooks et al. [33] presented the guarantee and time of norm emergence of their approaches in fully connected networks. Delgado [29] analyzed some conditions that could affect the convergence of his approach. Mihaylov et al. [34] and Leung et al. [28] presented the guarantee of norm emergence of their approaches in any networks, while the time was experimentally studied. Different from all above studies, this paper offers theoretical analysis about the guarantee and time of norm emergence in any networks.

### 6.3. Measurement of Whether a Norm Emerged

A widely used 90% threshold measurement [35] indicated that a norm emerges when at least 90% of agents adopt the same strategy. Villatoro et al. [36] argued that the threshold should be 100% to avoid the possibility that agents might deviate from an emerged norm. In this paper, we use the 100% threshold because in IIoT environments, if the 90% threshold is used, some agents might consistently encounter conflicts, which is undesired by the agents, reducing system scalability.

### 6.4. Networks

Networks are widely used to determine which agents could interact with each other. Early work on norm emergence considered only fully connected networks where an agent could interact with all other agents [1,5,37]. Later, Delgado [29] studied norm emergence in more realistic small-world and scale-free networks, which are mostly studied in current literature. Recently, community networks receoved increasing attention due to their wide existence [17,32]. All these types of networks, however, were static where the connections between agents did not change. Some studies [22,34] involved dynamic networks, but the changes in connections happened randomly without realistic restrictions. In this paper, we not only use the four popular types of static networks but also develop a vehicle movement simulator to mimic IIoT environments. The simulator models various traffic situations where the connections between agents are continuously changing and the changes are restricted by factors like the road structure. The simulator allows us to model more complex interactions, helping study norm emergence under more representative IIoT applications.

## 7. Conclusions and Future Work

In this paper, we proposed a decision-making framework for agents to solve the problem of norm emergence through conflict-blocking interactions, specifically in the context of IIoT. Both theoretical and experimental analyses were conducted to demonstrate the effectiveness of the proposed framework in addressing the unique challenges of IIoT environments: lack of global visibility, system scalability, and agent heterogeneity.

Some potential directions for future work were mentioned in the analysis of experiments, which include (1) adaptively setting the help-checking interval τ for faster norm emergence; (2) ensuring that the number of agents has a linear or near-linear influence on the time of norm emergence; (3) enabling a large number of agents to adopt the same strategy quickly to expedite norm emergence; (4) theoretically analyzing the standard deviation of the time of norm emergence; and (5) updating the vehicle movement simulator to cover more realistic IIoT environments.

## Figures and Tables

**Table 1:** Two examples of 2-player 2-strategy coordination games (payoff of the row player is shown first).

(**a**)		
	Go	Give Way to Vehicles on Right
Give way to vehicles on left	2, 3	−1, −1
Go	−2, −2	3, 2
(**b**)		
	Right	Left
Right	1, 1	−1, −1
Left	−1, −1	1, 1

**Table 2:** The meaning of abbreviations and symbols used across sections.

MAS	a multi-agent system
PSNE	a pure-strategy Nash equilibrium of a coordination game (see Section 2.1)
ASCS, HSOS	two preferred strategy selection methods that can be used by agents in conflict resolution (see Section 3.4)
MS	the majority strategy, which is adopted by most agents in an MAS
SLMS	selecting local majority strategy as a preferred strategy, which is a part of HSOS
SCS	selecting current strategy as a preferred strategy, which is a part of HSOS
$R_{s}$	the rate of selecting MS as a preferred strategy
$R_{a}$	the rate of adopting MS for conflict resolution
lc	the local conformity of an agent (see Definition 8)
*n*	the number of agents in an MAS
*m*	the number of strategies of a coordination game
μ	the proportion of agents who adopt MS
*h*	the proportion of agents who apply HSOS

## Data Availability

The data generated during this study are available upon request.
